# Efficacy of low-dose methimazole in control of multiple relapses of Graves’ hyperthyroidism: a case report

**DOI:** 10.1186/s13256-021-02788-4

**Published:** 2021-04-23

**Authors:** Fereidoun Azizi, Atieh Amouzegar, Hengameh Abdi

**Affiliations:** Endocrine Research Center, Research Institute for Endocrine Sciences, Shahid Beheshti University of Medical Sciences, No 24, Aerabi St, Daneshjoo Blv, Velenjak, Tehran, Iran

**Keywords:** Graves’ disease, Hyperthyroidism, Methimazole, Relapse, Case report

## Abstract

**Introduction:**

Methimazole (MMI) is the treatment of choice for patients with Graves’ disease. The major drawback of this treatment is the relapse of hyperthyroidism in half of the patients after discontinuation of the recommended conventional 12–18 months of MMI treatment. TSH receptor antibody (TRAb) concentration is recognized as the strongest predictor of hyperthyroidism relapse. In this case report, efficacy of low-dose MMI to control hyperthyroidism even after multiple recurrences in the setting of normal TRAb concentrations is shown.

**Case presentation:**

An 80-year-old Iranian woman with Graves’ disease was treated with MMI for 31 years. While receiving treatment, she always had a normal serum TRAb concentration; however, three times during the 31 years she decided to stop MMI therapy, and each time the disease recurred 16–21 months after MMI withdrawal. It is noteworthy that she maintained euthyroidism with the low-dose 1.25–2.5 mg MMI daily without any adverse events during three decades of treatment.

**Conclusions:**

Normal serum TRAb is not a sufficiently strong marker to predict relapse of Graves’ hyperthyroidism. Long-term therapy with low-dose MMI is an effective and safe treatment to sustain euthyroidism.

## Introduction

Graves’ disease is an autoimmune condition caused by thyrotropin (thyroid stimulating hormone [TSH]) receptor stimulation via TSH receptor antibodies (TRAb) with a lifetime incidence rate of 0.5–3%. The majority of patients with Graves’ hyperthyroidism have a prolonged course characterized by episodes of remission and relapse for many years [1]. There are three forms of therapeutic approaches, namely, antithyroid drugs (ATDs), radioiodine (RAI) and surgery, but none are able to cure the disease in all patients [2]. In recent years, ATDs and in particular methimazole (MMI) or carbimazole, have become the first treatment of choice for Graves’ disease [3, 4]; however, relapse of hyperthyroidism occurs in almost half of the patients after withdrawal of the conventional 12–18 month ATD therapy [5]. Two systematic reviews/meta-analyses have shown that long-term continuous MMI is an effective and safe therapeutic mode for durable euthyroidism in Graves’ hyperthyroidism [6, 7]; in addition, the majority of such patients enter remission following discontinuation of long-term ATD treatment [8, 9].

Many factors are associated with the relapse of Graves’ hyperthyroidism, of which serum TRAb concentration has the strongest effect; other recognized factors include biochemically severe disease, goiter size, smoking, postpartum period, Graves’ orbitopathy and prolonged treatment [2, 10]. However, none of these factors are sufficiently strong markers to predict relapse in an individual patient.

We report here a case of Graves’ disease that responded well to low maintenance dose of MMI, with normalization of serum TRAb concentration but multiple relapses of hyperthyroidism upon discontinuation of MMI, even following long-term MMI treatment.

## Case presentation

**Fig. 1:**
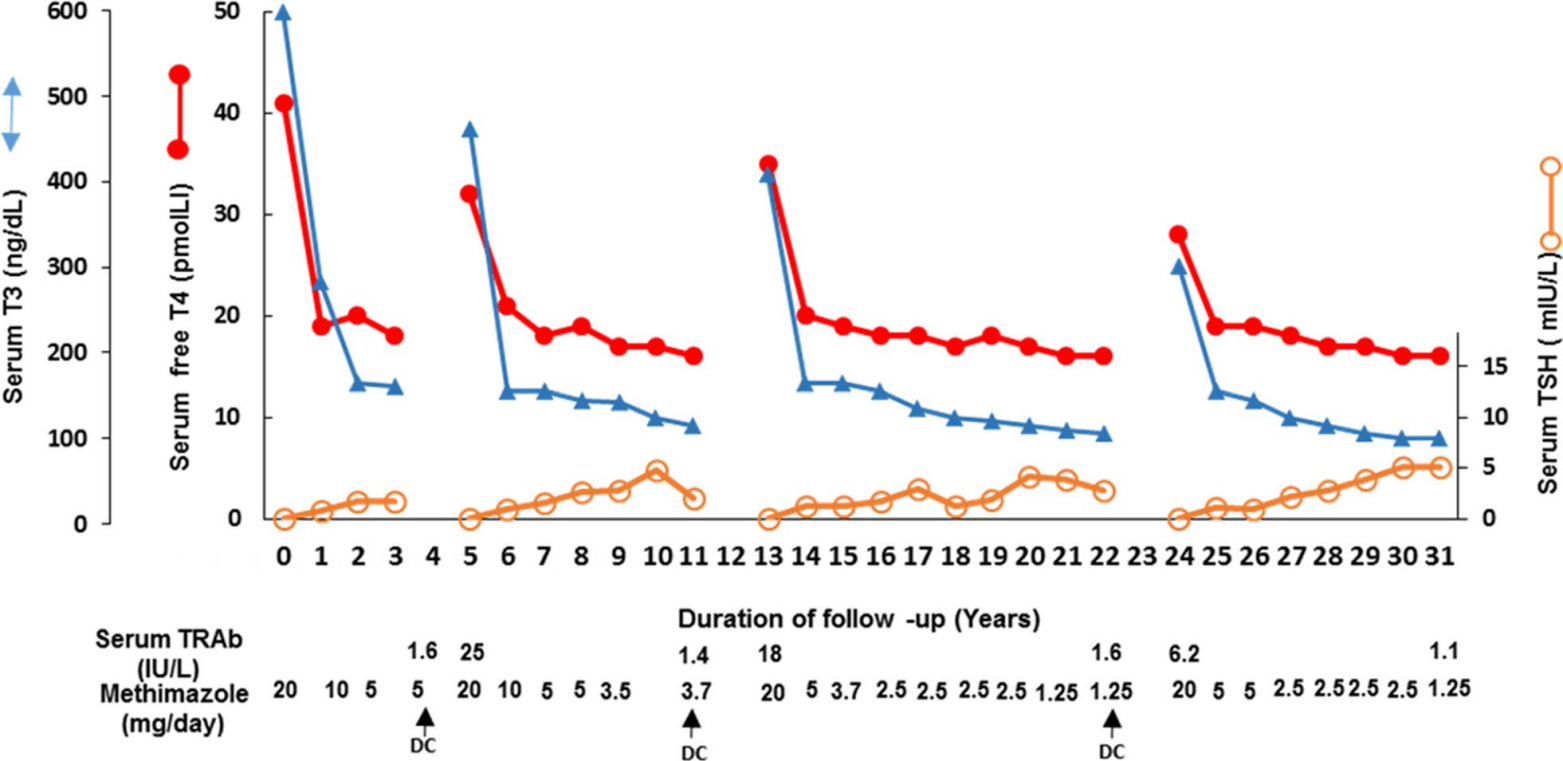
Patient’s response to methimazole (MMI) treatment following three recurrences of Graves’ hyperthyroidism. She stopped treatment on three occasions; after each time, hyperthyroidism recurred but was appropriately managed by re-initiating MMI therapy, with subsequent normalization of serum free thyroxine (*fT4*), triiodothyronine (*T3*), thyroid stimulating hormone (*TSH*) and TSH receptor antibody (*TRAb*) each time. The metimazole dose gradually decreased to a minimum of 1.25 mg daily

A 49-year-old married woman firstly consulted the medical center in July 1988 because of palpitation and anxiety. The symptoms had started the previous May and she had lost 2 kg in the meantime. Past medical and family histories were unremarkable. She was a never-smoker. Physical examinations revealed a blood pressure of 140/70 mm/Hg, pulse rate of 106 bpm, lid lag, staring gaze without proptosis, diffuse goiter of around 35 g, and hand tremors. Laboratory test results revealed serum free thyroxine (fT4) at 40 pmol/L (reference range 9–23 pmol/L), total triiodothyronine (T3) of 620 ng/dL (reference range 80–200 ng/dL) and TSH of < 0.01 mU/L. She was started on methimazole (MMI) 20 mg daily and was visited monthly for the first 3 months and every 3–6 months thereafter. At each visit the dose of MMI was adjusted to maintain fT4 and T3 concentrations in the mid-normal values. Serum TSH was 0.8 mU/L 1 year after the start of treatment. She felt well and stayed on MMI treatment for 3 years. In June 1991, she was on MMI 5 mg daily; at this time thyroid function test results (TFTs) were: fT4 = 19.0 pmol/L, T3 = 180 ng/dL, TSH = 2.1 mU/L, and serum TRAb concentration was 1.6 IU/L (assay cutoff point for diagnosis of Graves’ disease: 1.75 IU/L). MMI was discontinued and she stayed euthyroid until March 1993 when she experienced weight loss, tachycardia, and tremor; at this time, fT4 = 31.5 pmol/L, T3 = 460 ng/dL, TSH < 0.002 mU/L and TRAb = 25 IU/L. She was re-started on MMI 20 mg daily with the titration method and stayed euthyroid for 6 years. In September 1999, she had normal TFTs and serum TRAb was 1.4 IU/L and decided to discontinue MMI treatment. She stayed euthyroid until April 2001 when she returned to the medical center with typical symptoms and signs of hyperthyroidism, elevated serum fT4 and T3 levels and suppressed TSH level, with serum TRAb level of 18 IU/L. MMI treatment was re-started once again, and she stayed euthyroid for another 9 years, with serum TSH of 0.6–2.4 mU/L (Fig. 1). The dose of MMI was gradually decreased to 2.5 mg daily to prevent an increase in serum TSH concentration. In October 2010, she was euthyroid on 1.25 mg MMI daily, with serum TRAb of 1.6 IU/mL, and she decided once again to discontinue MMI. Hyperthyroidism recurred in February 2012, with a serum TRAb of 6.2 IU/L. At present, she has been taking MMI for last 7 years and has maintained euthyroidism. She was last seen in September 2019 at the age of 80 years; at this time the TFTs were: serum fT4 = 15.2 pmol/L, total T3 = 129 ng/dL and TSH = 4.8 mU/L. She was on 1.25 mg MMI daily and wanted to continue MMI lifelong. During the 31 years of MMI treatment, no side effects were reported. She was on atorvastatin 10 mg daily, and yearly routine laboratory test measurements, including cell count, lipid profile, liver enzymes, serum creatinine, and urinalysis, were within normal limits. Throughout the years of treatment, she refused RAI as an alternative therapeutic choice; she also refused thyroid surgery, suggested after the second relapse.

## Discussion and conclusions

The present case report demonstrates the safety and efficacy of the long-term MMI treatment in a Graves’ patient with multiple episodes of recurrence. A few specific findings in this patient should be discussed: (1) multiple recurrences of hyperthyroidism after 3, 6 and 9 years of continuous MMI treatment; (2) normal serum TRAb concentrations at the third, 11th and 22nd years of treatment, which did not predict relapse of hyperthyroidism; and (3) the efficacy of low-dose MMI to maintain euthyroidism for many years after each relapse.

The natural history of Graves’ disease consists of episodes of remission and relapse of hyperthyroidism for many years [2, 5]. It has been reported that 30–70% of patients stay euthyroid after the first course of treatment with ATDs and that the relapse rate of hyperthyroidism reaches a plateau at 4 years after drug withdrawal. However, recurrence of hyperthyroidism may occur even 30–40 years after the first presentation.

Serum concentration of TRAb is considered to be the strongest predictor of relapse of hyperthyroidism [10]. Studies have shown that those with persistent high TRAb levels during ATD treatment have 80–100% chance of recurrence and that most patients (70–80%) with undetectable serum TRAb concentration stay in remission following discontinuation of therapy. The rate of remission in patients with normal serum TRAb concentration has been estimated to be about 36%, indicating that normal TRAb concentration does not predict cure of the disease [2, 11]. In our patient, serum TRAb concentration remained < 1.75 IU/L (assay cutoff point for Grave’s disease) and specifically was 1.6, 1.4 and 1.6 IU/L before each of the three times she withdrew from MMI treatment; however, hyperthyroidism still recurred 16–20 months after treatment discontinuation.

During conventional MMI treatment for 12–18 months, the recommended MMI dosage is 5–40 mg at treatment initiation followed by a gradual decrease (titration method) to maintain euthyroidism [2]. A daily dose of 2.5–10 mg MMI maintains euthyroidism until the end of treatment. Long-term administration of MMI has been shown to be effective [6] and safe [7] in Graves’ patients, in particular in those who have recurrence of hyperthyroidism after the conventional 12–18 months of treatment [12]. It has been shown that the required dosage of MMI declines throughout years of continuous long-term MMI treatment [9]. In the patient reported here, 1.25 mg MMI has been effective in maintaining euthyroidism. The authors have made a similar observation over the years in clinical practice, noting that in many patients receiving continuous MMI treatment, weekly administration of two tablets of 5 mg MMI was sufficient to maintain euthyroidism and its discontinuation was accompanied by relapse of hyperthyroidism within few months (details not reported here).

In 1979, Singerland and Burrows stated that very long-term administration of ATD is safe [13], and recent studies have confirmed that continuous long-term MMI therapy is one of the most appropriate methods to control of hyperthyroidism in Graves’ disease [6, 8, 9, 14].

In conclusion, normal serum TRAb concentration is not a sufficiently strong diagnostic marker to predict relapse of Graves’ hyperthyroidism. Long-term therapy with low-dose MMI is effective and safe to sustain euthyroidism.

## Data Availability

Nearly all relevant data are presented in the manuscript.

## Abbreviations

ATD: Antithyroid drug
fT4: Free thyroxine
MMI: Methimazole
RAI: Radioactive iodine
T3: Triiodothyronine
TRAb: TSH receptor antibody
TSH: Thyroid stimulating hormone
